# Matrix Metalloproteinase Gene Activation Resulting from Disordred Epigenetic Mechanisms in Rheumatoid Arthritis

**DOI:** 10.3390/ijms18050905

**Published:** 2017-04-25

**Authors:** Yasuto Araki, Toshihide Mimura

**Affiliations:** 1 Department of Rheumatology and Applied Immunology, Faculty of Medicine, Saitama Medical University, Saitama 350-0495, Japan; toshim@saitama-med.ac.jp; 2 Project Research Division, Research Center for Genomic Medicine, Saitama Medical University, Saitama 350-0495, Japan

**Keywords:** matrix metalloproteinase, rheumatoid arthritis, epigenetics, gene transcription

## Abstract

Matrix metalloproteinases (MMPs) are implicated in the degradation of extracellular matrix (ECM). Rheumatoid arthritis (RA) synovial fibroblasts (SFs) produce matrix-degrading enzymes, including MMPs, which facilitate cartilage destruction in the affected joints in RA. Epigenetic mechanisms contribute to change in the chromatin state, resulting in an alteration of gene transcription. Recently, MMP gene activation has been shown to be caused in RASFs by the dysregulation of epigenetic changes, such as histone modifications, DNA methylation, and microRNA (miRNA) signaling. In this paper, we review the role of MMPs in the pathogenesis of RA as well as the disordered epigenetic mechanisms regulating MMP gene activation in RASFs.

## 1. Introduction

Proteases are enzymes that catalyze the hydrolysis of peptide bonds in polypeptide chains of target proteins. They stringently regulate numerous essential physiological processes, including autophagy, cellular protein degradation (through both lysosomes and the ubiquitin–proteasome system), the immune response, cell death, and signal transduction [1,2,3,4]. The dysregulation of protease activity can result in the development of a number of diseases, such as inflammatory diseases, cardiovascular diseases, cancer, and neurological diseases [5,6,7,8]. Therefore, several small molecules that specifically inhibit particular proteases have been developed as effective drugs [9].

To date, approximately 600 proteases have been identified in humans [10]. Proteases are divided into endopeptidases and exopeptidases. The endopeptidases, also called proteinases, hydrolyze internal peptide bonds of polypeptide chains. The exopeptidases include carboxypeptidases and aminopeptidases that cleave substrates at the carboxyl- and amino-termini of polypeptide chains, respectively. Based on the mechanisms of proteolysis, endopeptidases are grouped into five major classes, including aspartic, cysteine, threonine, serine, and metalloproteinases [11,12]. Aspartic, cysteine, and threonine proteinases are involved in intracellular pathways, whereas serine and metalloproteinases are associated with extracellular pathways. The metalloproteinases include matrix metalloproteinases (MMPs), which are involved in the pathogenesis of numerous diseases, such as rheumatoid arthritis (RA).

The mechanisms regulating MMP transcription have been clarified. Pro-inflammatory cytokines, including tumor necrosis factor-α (TNF-α) and interleukin (IL)-1β, activate MMP genes through the binding of several different transcription factors (TFs), such as mitogen-activated protein kinase (MAPK), nuclear factor-κB (NF-κB), and activator protein-1 (AP-1), to their promoters [13,14,15]. IL-6 is another pro-inflammatory cytokine that is associated with the pathogenesis of RA [16]. Recently, IL-6 was also reported to induce MMP gene activation through the binding of signal transducer and activator of transcription (STAT) 3 to MMP promoters in RA synovial fibroblasts (SFs) [17]. Epigenetic changes have been shown to affect chromatin structure without a change in DNA sequence itself. Therefore, both the chromatin state and the accessibility of TFs are thought to lead to aberrant gene transcription in RA. There is evidence that epigenetic mechanisms, such as histone modifications, DNA methylation and microRNA (miRNA) expression, contribute to MMP gene activation in several diseases, including RA.

## 2. The Structure and Function of Matrix Metalloproteinases

**Figure 1:**
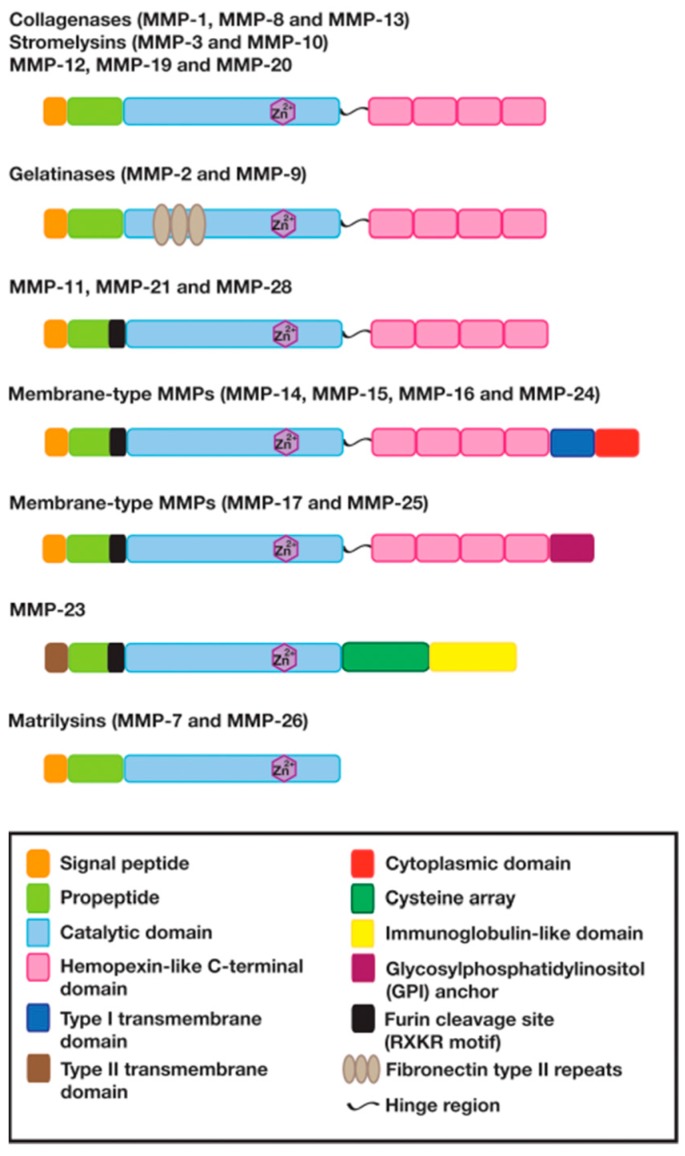
Matrix metalloproteinases (MMPs) are comprised of different subdomains. Most have a minimal domain (a signal peptide, propeptide, and catalytic domain), a hinge region, and a hemopexin-like C-terminal domain. Gelatinases (MMP-2 and MMP-9) contain three repeats of a fibronectin type II motif in the catalytic domain. Membrane-type MMPs have a type I transmembrane domain/cytoplasmic domain or a glycosylphosphatidylinositol (GPI) anchor in addition. MMP-23 contains a type II transmembrane domain, a cysteine array, and an immunoglobulin-like domain. The propeptide contains a furin cleavage site (RXKR motif) in MMP-11, MMP-21, MMP-23, MMP-28, and membrane-type MMPs. MMP-7 and MMP-26 lack both a hinge region and a hemopexin-like C-terminal domain.

MMPs are a family of zinc-dependent metalloendopeptidases that play a critical role in the turnover of a variety of extracellular matrix (ECM) proteins [13,18]. The human MMP family currently consists of 23 members and is subdivided into five major subfamilies according to functional and structural properties: (1) the collagenases (MMP-1, MMP-8, and MMP-13); (2) the gelatinases (MMP-2 and MMP-9); (3) the stromelysins (MMP-3, MMP-10, and MMP-11); (4) a heterogenous subgroup, such as matrilysins (MMP-7 and MMP-26), enamelsin (MMP-20), macrophage metalloelastase (MMP-12), and others (MMP-19, MMP-21, MMP-23, MMP-27, and MMP-28); and (5) the membrane-type (MT)-MMPs—MMP-14 (MT1-MMP), MMP-15 (MT2-MMP), MMP-16 (MT3-MMP), MMP-17 (MT4-MMP), MMP-24 (MT5-MMP), and MMP-25 (MT6-MMP)—, (Table 1) [19,20]. Substrates for the MMPs include most of the ECM components, such as collagen (types I, II, III, IV, V, VI, VII, VIII, IX, X, XI, and XIV), aggrecan, elastin, fibronectin, gelatin, and laminin. MMP-1, MMP-3, MMP-8, MMP-10, MMP-12, MMP-13, MMP-19, and MMP-20 contain several distinct subunits, such as a minimal domain, hinge region, and hemopexin-like C-terminal domain (Figure 1) [21,22]. The minimal domain is comprised of a signal peptide, a propeptide, and a catalytic domain. The signal peptide is cleaved by signal peptidases when the MMPs move to the endoplasmic reticulum. The propeptide contains a cysteine residue and a furin cleavage site (Arg–X–Lys–Arg motif) in MMP-11, MMP-21, MMP-23, MMP-28, and MT-MMPs. The interaction of the cysteine residue with the zinc ion of the catalytic domain renders the MMPs inactive. Pro-protein converting enzymes, such as furin, make the enzymes active by disrupting this interaction (this process is known as a cysteine switch). The hinge region links the catalytic domain to the hemopexin-like C-terminal domain. In the gelatinases (MMP-2 and MMP-9), three repeats of a fibronectin type II motif exist in the catalytic domain and enable them to bind and degrade gelatin. The hemopexin-like C-terminal domain, which is composed of four repeats that resemble hemopexin, determines substrate specificity. The hinge region and hemopexin-like C-terminal domain are absent from the matrilysins (MMP-7 and MMP-26). MMP-23 lacks a signal peptide and contains a type II transmembrane domain, a cysteine array, and an immunoglobulin-like domain. MT-MMPs are inserted in the plasma membrane by a type I transmembrane domain with a cytoplasmic domain (MMP-14, MMP-15, MMP-16, and MMP-24) or by a glycosylphosphatidylinositol (GPI) anchor (MMP-17 and MMP-25) [23].

## 3. The Pathogenesis of Rheumatoid Arthritis

### 3.1. The Epidemiology and Etiology of RA

RA is a chronic systemic inflammatory disorder that is caused by an autoimmune response, which is characterized by the presence of anti-citrullinated protein/peptide antibodies (ACPA) and rheumatoid factors (RF) [24,25,26,27]. RA affects approximately 1% of the population worldwide and is two-to-three-fold more common in women than in men [28]. Recently, new treatments, such as antirheumatic biologics and Janus kinase (JAK) inhibitors, have been developed and have revolutionized RA therapy [29,30,31]. Nevertheless, randomized controlled clinical trials have demonstrated that these biologic drugs can only treat approximately 50% of RA patients successfully on average. They work via the following mechanisms: (1) TNF-α inhibition (infliximab [32,33,34,35], etanercept [36,37,38,39,40,41], adalimumab [42,43,44], golimumab [45,46,47,48,49], and certolizumab pegol [50]) [51,52,53]; (2) B cell depletion (rituximab [54,55]); (3) disruption of T cell co-stimulation (abatacept [56,57,58,59,60,61]); (4) IL-1 inhibition (anakinra [62]); and (5) IL-6 inhibition (tocilizumab [63,64,65,66,67,68]). The remainder of RA patients do not respond or do not fully benefit from these treatments. The therapeutic effect of the JAK inhibitor tofacitinib is similar to these antirheumatic biologics [69,70,71,72,73].

The pathogenesis of RA is multifactorial and has not yet been completely elucidated [74]. Recent advances have demonstrated that environmental factors trigger RA in genetically predisposed individuals, evidence for which includes a higher concordance for RA in monozygotic twins than in either dizygotic twins or non-twins [75,76,77], the high relative risk for RA in multiplex families [78], the association of susceptibility to RA with human leukocyte antigen (HLA)-DRB1 alleles that contain a shared epitope (which is associated with RA severity and the production of ACPA in RA) [79,80,81], and a number of RA risk genes identified by genome-wide association studies (GWAS), such as PTPN22 (protein tyrosine phosphatase, non-receptor type 22), STAT4, CCR6 (chemokine receptor 6), TNFAIP3 (TNF-α-induced protein 3), PADI4 (peptidyl arginine deiminase 4), CD40 (cluster of differentiation 40), and FCRL3 (Fc receptor-like protein 3) [82,83]. Conversely, certain environmental factors, including cigarette smoking [84,85], Epstein–Barr virus (EBV) infection [86,87], periodontitis caused by *Porphyromonas gingivalis* [88,89], sex hormones [90,91], vitamin D deficiency [92,93], and silica exposure [94], have been shown to be associated with the pathogenesis of RA. In addition, recent advances have indicated that epigenetic mechanisms are associated with the etiology of RA [95,96].

### 3.2. The Pathological Roles of MMPs in RA

RA is characterized by progressive joint destruction with loss of bone and cartilage as well as the aggressive activation of SFs bearing a tumor-like appearance [97,98,99]. MMPs play a critical role in the pathogenesis of RA [20]. RASFs secrete various proteases, including MMPs that degrade ECM components, mainly proteoglycans and collagens, of articular cartilage in the affected joints [100]. Since MMP-1, MMP-3, MMP-9, and MMP-13 expression is upregulated in RASFs, MMPs are considered to play a critical role in the degeneration of cartilage in RA joints [17]. MMP-1 (collagenase 1) and MMP-13 (collagenase 3) cleave collagens, whereas MMP-3 (stromelysin 1) and MMP-9 (gelatinase B) target proteoglycans that are comprised of aggrecan. The degeneration of proteoglycans at the surface and the subsequent degradation of collagen fibrils in the deep zone together result in the destruction of articular cartilage. MMPs may thus play a distinct role in joint destruction in RA.

MMPs are potent markers for predicting the functional and radiographic outcome of joints in RA. The serum concentrations of MMP-1 and MMP-3 correlate with disease activity and predict the progression of joint destruction in RA [101,102,103]. Treatment with disease-modifying antirheumatic drugs (DMARDs) and antirheumatic biologics significantly downregulates the serum levels of MMP-1 and MMP-3 in parallel with the reduction in disease activity [104,105].

It has been shown that MT1-MMP (MMP-14) is the major MMP that is associated with cartilage invasion by RA synovial tissues/RASFs. MT1-MMP is highly expressed at the junction of pannus and cartilage and is responsible for the invasion of RASFs into cartilage [106]. MT1-MMP is involved in not only ECM remodeling but also the angiogenic response that plays an important role in the aggressive phenotype of RASFs [107]. Treatment with an MT1-MMP selective inhibitory antibody suppresses both cartilage destruction and disease progression in a collagen-induced arthritis (CIA) mouse model [108].

Several studies have focused on the phenotype of MMP-deficient mice. *MMP-8*-deficient mice have been backcrossed onto a Fas-defective MRL/lpr background that is characterized by spontaneous arthritis [109]. The mice showed earlier and more severe arthritis as well as an increase in neutrophil infiltration due to delayed apoptosis. Therefore, MMP-8 is considered to be anti-inflammatory. To examine whether gelatinases (MMP-2 and MMP-9) play an important role in RA, the development of antibody-induced arthritis in *MMP-2* or *MMP-9*-deficient mice has been investigated [110]. *MMP-2*-deficient mice show severe arthritis, whereas MMP-9-deficient mice exhibit mild arthritis. These findings suggest a suppressive role for MMP-2 and a pivotal role of MMP-9 in arthritis. *MMP-3*-deficient mice develop arthritis in a CIA model [111], and disruption of *MMP-3* gene does not prevent cartilage destruction.

Over the past decade, antirheumatic biologics and JAK inhibitors have been developed as effective drugs for RA. Because the agents still have several issues, including inadequate efficacy, infection risk, and cost, it has been expected that alternative therapies for RA will appear. MMPs are considered an important novel therapeutic target in RA because they are thought to be harmful in the disease. Pharmaceutical companies have thus synthesized small molecule MMP inhibitors [20], some of which have entered clinical trials for RA therapy [112,113]. For example, cipemastat, a selective inhibitor of collagenases (MMP-1, MMP-8, and MMP-13), is reportedly well tolerated with no serious adverse events [114,115]. It was expected to be a promising agent for RA therapy given results of animal models of arthritis [116,117]. However, the compound did not prevent progression of joint destruction in RA patients in phase III trials [118,119]. Clinical trials of all of other MMP inhibitors have also thus far been unsuccessful, and pharmaceutical companies have halted their development. There are several reasons why these clinical trials failed. Because MMP inhibitors target the catalytic sites of the enzymes, their specificity has been low. Furthermore, MMPs are not necessarily harmful in arthritic diseases. Some MMPs, including MMP-2 and MMP-8, exhibit protective effects against arthritic diseases as discussed above. At present, there are still several potentially good MMP inhibitors, such as a highly selective inhibitor of MMP-13 [120] and a humanized inhibitory antibody against MT1-MMP [108]. Further preclinical and clinical research is needed for the development of these novel inhibitors as useful therapeutics for RA.

The adamalysins, including ADAMs (a disintegrin and metalloproteinase) and ADAMTSs (ADAMs with thrombospondin motifs), are a family of membrane-associated zinc-dependent metallo exopeptidases that degrade ECM components [121]. Twenty-four ADAMs have been identified in humans and are implicated in reproductive processes. ADAM-17, known as TNF-α converting enzyme (TACE), cleaves the proform of TNF-α into its active form [122,123]. In addition, 18 ADAMTSs have been described in humans and are associated with pathological processes. ADAMTS-4 and ADAMTS-5/11 are aggrecanase-1 and aggrecanase-2, respectively, which are involved in cartilage destruction in arthritic diseases [124,125,126,127,128].

## 4. Epigenetic Mechanisms

### 4.1. Epigenetics and Chromatin Structure

Epigenetics is a relatively new area of research, first proposed by Waddington, in which gene regulation mediated by mutations modulates development. Subsequently, it was defined as a stably heritable phenotype resulting from changes in a chromosome without alterations in the DNA sequence [129]. Epigenetic changes are transmitted during the process of either mitosis or meiosis. Several major epigenetic mechanisms have been identified to date, including post-translational histone modifications, DNA methylation, and the expression of non-coding RNAs (ncRNAs) such as miRNAs [130]. These mechanisms determine the specific chromatin structure and consequently influence gene expression independently of the DNA sequence. Chromatin is a structure in which DNA is tightly packaged in the nuclei of eukaryotic cells [131]. The nucleosome is the fundamental subunit of chromatin and is composed of 147 bp of DNA wrapped around an octamer of the four core histones (H2A, H2B, H3, and H4). The chromatin state in DNA-regulating regions, such as promoters and enhancers, determines the accessibility for TFs and consequently affects gene transcription. The chromatin state is classified into two basic forms: euchromatin is an open state in which TFs can bind to promoters and activate genes, whereas heterochromatin is a closed state in which TFs are unable to reach promoters and genes are silent.

Genome-wide analyses of histone methylation and gene expression have elucidated four distinct forms of association, namely repressed, active, poised, and bivalent [132,133,134]. In repressed genes, heterochromatin suppresses gene transcription. In active genes, euchromatin activates gene transcription. In poised genes, euchromatin does not activate gene transcription while at rest; however, stimulation with certain factors such as cytokines induces gene activation rapidly. In bivalent genes, the chromatin contains both active and repressive histone markers. Chromatin is able to convert into euchromatin or heterochromatin either through cell differentiation or upon activation.

### 4.2. Histone Modifications

**Figure 2:**
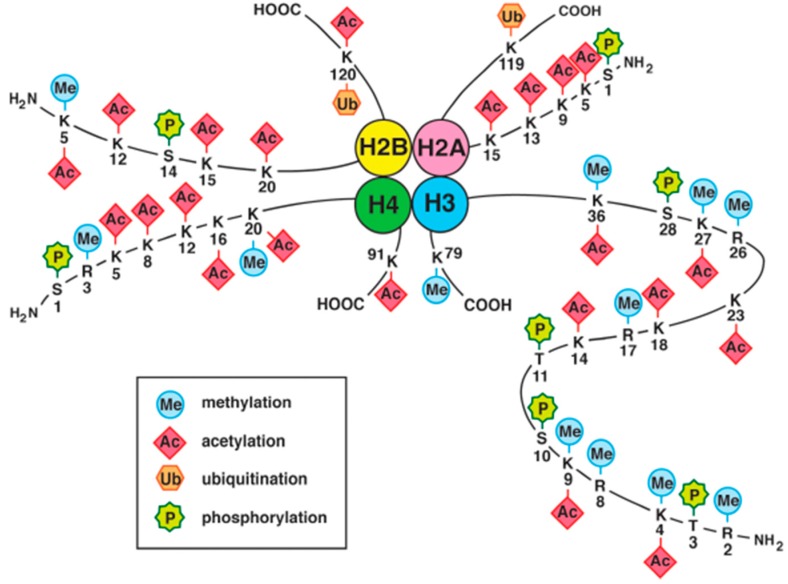
Histone modifications, including methylation, acetylation, ubiquitination, phosphorylation, and sumoylation have a variety of biological functions, including the regulation of chromatin states and gene transcription.

The histone code hypothesis that Strahl and Allis proposed means that multiple histone modifications, acting in a combinatorial or sequential fashion on one or multiple histone tails, specify unique downstream functions [135]. Histone tails are subject to covalent post-translational modifications, including methylation, acetylation, ubiquitination and phosphorylation, that regulate whether genes are in a transcriptionally active or silent chromatin state (Figure 2) [136,137,138,139]. The modifications are markers that reflect both the chromatin state and gene transcription status. Active histone markers, which are located on euchromatin and activate gene transcription, include acetylation—H2A lysine 5 (H2AK5), H2AK9, H2AK13, H2AK15, H2BK5, H2BK12, H2BK15, H2BK20, H2BK120, H3K4, H3K9, H3K14, H3K18, H3K23, H3K27, H3K36, H4K5, H4K8, H4K12, H4K16, H4K20, and H4K91—, methylation—H2BK5, H3 arginine 2 (H3R2), H3K4, H3R8, H3K9, H3R17, H3R26, H3K27, H3K36, H3K79, H4R3, and H4K20—, phosphorylation—H2A serine 1 (H2AS1), H2BS14, H3 threonine 3 (H3T3), H3S10, H3T11, H3S28, and H4S1—, and ubiquitination (H2AK119, H2BK120). Repressive histone markers, which exist on heterochromatin and suppress gene transcription, include methylation (H3K9, H3K27, and H4K20), ubiquitination (H2AK119), and sumoylation (H2AK126, H2BK6, and H2BK7). A complex combination of these histone modifications controls chromatin states and gene transcription. The regulation of the chromatin state by combinatorial patterns of histone acetylation and methylation has been demonstrated in a genome-wide analysis [140].

To date, histone methylation and acetylation have been well studied. Histone methyltransferases (HMTs) transfer methyl groups, whereas histone demethylases (HDMs) remove methyl groups [141,142]. HMTs and HDMs specifically catalyze particular lysine or arginine residues [143,144]. The functions of histone methylation are defined by both the methylated residue and the number of methyl groups [145,146]. Histone acetyltransferases (HATs) add acetyl groups to lysine residues, resulting in gene activation [147,148]. Histone deacetylases (HDACs) remove acetyl groups from lysine residues, leading to gene silencing [149,150].

Disordered histone modifications in MMP genes have been demonstrated in several diseases, such as cervical cancer and cardiovascular diseases. HDAC10 represses the expression of MMP-2 and MMP-9, which are involved in cancer cell invasion and metastasis [151]. The expression level of HDAC10 is low in patients exhibiting lymph node metastasis in cervical squamous cell carcinoma. In addition, HDAC10 binds to MMP-2 and MMP-9 promoters and decreases histone acetylation levels. Post-myocardial infarction induction of MMP-9 causes deleterious effects on the left ventricle and ECM remodeling in the myocardical infarction region [152]. MMP-9 upregulation is associated with an increase in histone acetylation by HATs.

### 4.3. DNA Methylation

**Figure 3:**
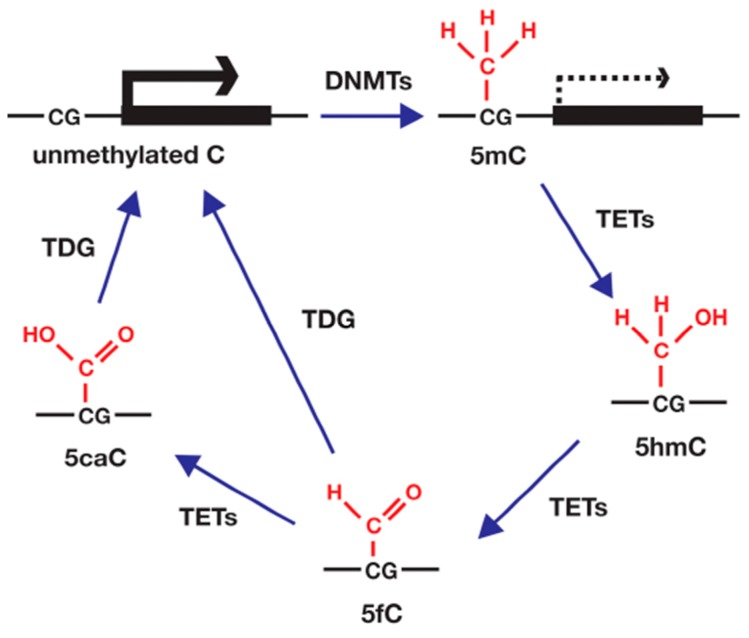
DNA methylation and demethylation are catalyzed by DNA methyltransferases (DNMTs) and ten-eleven translocation (TET) enzymes, respectively. Cytosine at CpG sites is methylated by DNMTs to yield 5-methylcytosine (5mC). The 5mC is hydroxylated by TET enzymes to yield 5-hydroxymethylcytosine (5hmC), which in turn is oxidized to 5-formylcytosine (5fC) and 5-carboxylcytosine (5caC). The 5fC and 5caC are converted to unmethylated C by thymine DNA glycosylate (TDG)-mediated base excision repair.

DNA methylation is a process in which the carbon at the 5′ position of cytosine is methylated (5mC) (Figure 3). DNA methylation in mammals is generated in CpG dinucleotides that are generally clustered in the promoters of genes (CpG islands). A high degree of DNA methylation blocks the association of TFs and results in gene silencing, whereas a lack of DNA methylation permits TF binding and leads to gene activation. It is possible that DNA methylation in other regions, such as the gene body, is associated with an increase in gene expression. DNA methylation also plays an important role in suppressing the expression of potentially harmful transposable and viral elements in genomes. DNA methylation is catalyzed by DNA methyltransferases (DNMTs), including de novo methyltrasferases (DNMT3A, DNMT3B) and a maintenance methyltransferase DNMT1. The former enzymes add a methyl group to unmethylated or hemimethylated CpG sites, giving rise to new DNA methylation regions. The latter enzyme adds a methyl group to hemimethylated CpG sites in daughter strands during cell division, maintaining the methylation patterns throughout the course of cell replication. Although DNA methylation was generally considered stable, recent studies have shown that methylation can be erased by both passive and active mechanisms. Passive DNA demethylation is caused by a decrease in DNMT activity during cell division, which gradually reduces methylation. Active DNA demethylation is catalyzed by the ten-eleven translocation (TET) family of enzymes (TET1, TET2, and TET3), which convert 5mC into 5-hydroxymethylcytosine (5hmC). Further, TET enzymes exchange 5hmC for 5-formylcytosine (5fC) and 5-carboxylcytosine (5caC). An unmethylated cytosine is generated either through a replication dependent dilution of 5hmC or the removal of either 5fC or 5caC by thymine DNA glycosylate (TDG)-mediated base excision repair.

Disordered DNA methylation in MMP genes has been reported in several diseases, such as diabetes, cerebral ischemic stroke, and periapical inflammation. Activated MMP-9 is involved in the development of diabetic retinopathy [153]. In the MMP-9 promoter in diabetic retinal endothelial cells, 5mC levels are decreased, whereas TET2 binding and 5hmC levels are increased. MMP-2 activity is increased in cerebral ischemic stroke [154]. DNA methylation levels of the MMP-2 gene in the peripheral blood of patients with stroke are lower than in controls. High MMP-9 mRNA expression and low DNA methylated profiles of the MMP-9 gene have been found in periapical inflammatory lesions [155]. This indicates a critical role for DNA methylation in periapical inflammation.

### 4.4. miRNAs

**Figure 4:**
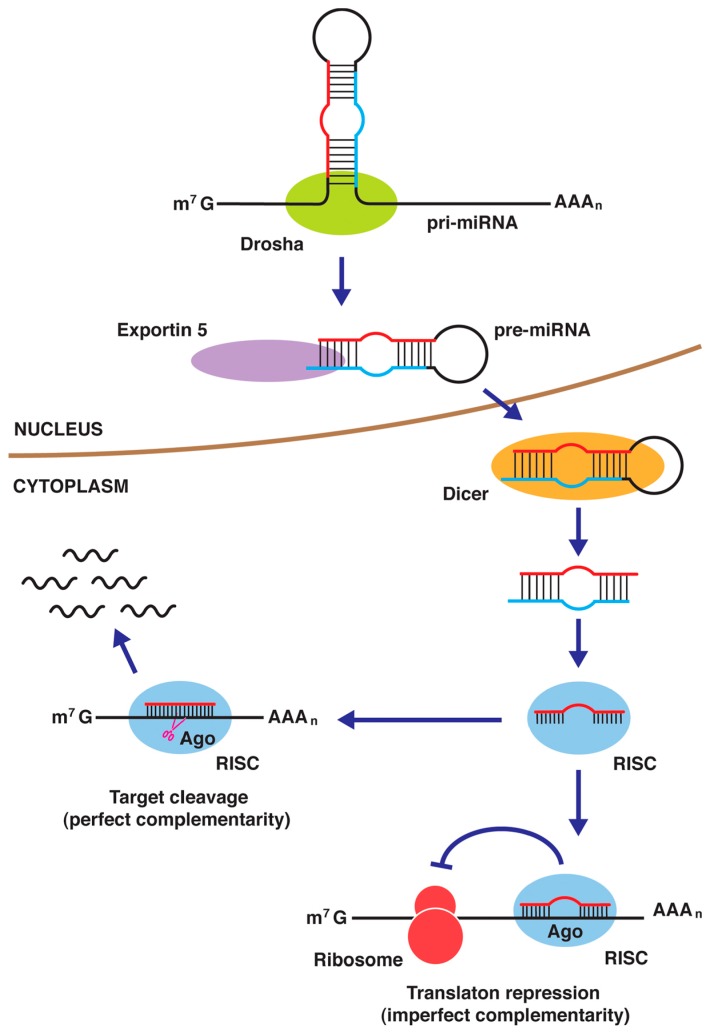
MicroRNAs (miRNAs) are initially transcribed as long primary miRNA (pri-miRNA) and cleaved by Drosha to yield a short precursor miRNA (pre-miRNA) in the nucleus. After export to the cytoplasm, the pre-miRNA is processed by Dicer to yield a double stranded-miRNA complex. Following the association with the RNA-induced silencing complex (RISC) and the removal of the complementary strand, a mature miRNA cleaves a target mRNA or represses its translation. Ago: Argonaute; m^7^ G: RNA 7-methylguanosine cap.

Mature miRNAs are short single-stranded ncRNAs (19–25 nucleotides) that are associated with one or more mRNAs and cause gene silencing through mRNA cleavage or translational repression (Figure 4) [156,157,158]. miRNAs are encoded in the genome and are transcribed to long primary miRNAs (pri-miRNAs) of several kb in length by RNA polymerase II. Several miRNA genes exist as clusters and are generated from common pri-miRNAs. In the nucleus, Drosha processes the pri-miRNAs into 60–80-nucleotide short precursor miRNAs (pre-miRNAs) that have a hairpin structure. Alternatively, a few miRNAs are present in introns (known as “mitrons”), which become lariat products after splicing. A lariat-debranching enzyme liberates the resulting products to yield pre-miRNAs. The nuclear export protein exportin 5 (EXP-5) exports pre-miRNAs to the cytoplasm. In the cytoplasm, the RNase III-type enzyme Dicer (DCR-1) cleaves the pre-miRNAs to generate a duplex containing two guide and passenger strands, termed miRNA and miRNA*, respectively. The double-stranded miRNA complex binds to the RNA-induced silencing complex (RISC) that contains Argonaute (Ago1). Mature miRNAs become functional after the complementary strands are removed from the RISC. Some miRNAs are derived from the guide strands, and others are produced from the passenger strands. Perfect complementarity between miRNAs and the mRNA targets results in the cleavage of the mRNA strands, whereas imperfect complementarity leads to translational repression.

Disordered miRNA expression that leads to aberrant MMP gene expression has been shown in several diseases. Downregulation of miR-29b-3p increases MMP-2 expression in arterial calcification [159]. Downregulation of miR-376c enhances MMP-2 expression in prostate cancer bone metastasis [160]. Decreases in miR-133a, miR-27b, and miR-155 upregulate MMP-9, MMP-13, and MMP-16 expression, respectively, resulting in intervertebral disc degeneration [161,162,163]. Reduced miR-106b expression causes breast cancer cell invasion through the upregulation of MMP-2 [164].

## 5. Epigenetic Mechanisms Regulating MMP Transcription in RA

### 5.1. Disordered Histone Modifications in MMP Genes in RA

Disordered histone methylation gives rise to active chromatin states in MMP genes in RASFs. The active histone marker H3K4me3 increases, while the repressive histone marker H3K27me3 decreases in the promoters of MMP-1, MMP-3, MMP-9 and MMP-13 in RASFs [17]. WD (Trp–Asp) repeat domain 5 (WDR5) is a core subunit of complex proteins associated with SET1 (Su(var)3-9, Enhancer-of-zeste and Trithorax 1) (COMPASS) as well as COMPASS-like complexes that methylate H3K4 [165]. *WDR5* knockdown downregulates not only the levels of H3K4me3, but also the expression of MMP-1, MMP-3, MMP-9 and MMP-13 in RASFs; H3K4me3 has been suggested to regulate spontaneous MMP gene transcription in RASFs. IL-6 signaling increases the expression of MMP-1, MMP-3 and MMP-13, but not MMP-9, despite the active chromatin states in all of these promoters in RASFs. The IL-6-induced transcription factor STAT3 is associated with the MMP-1, MMP-3 and MMP-13 promoters, but not the MMP-9 promoter. It has been suggested that the difference in response to IL-6 signaling in MMP genes is caused by STAT3 binding to their promoters. The binding of STAT3 to MMP-1, 3 and 13 promoters upregulates gene transcription, whereas the inaccessibility of STAT3 in the MMP-9 promoter does not allow gene activation. Aberrant histone methylation and the binding of STAT3 to the promoters differentially regulate constitutive and IL-6-induced gene expression of MMP-1, MMP-3, MMP-9 and MMP-13 in RASFs.

It is possible that disordered histone-modifying enzymes, including HMTs, HDMs, HATs and HDACs, disrupt histone modifications in RA. An H3K27-catalyzing HMT, enhancer of zeste homologue 2 (EZH2), is highly expressed in RASFs and induced by TNF-α through the NF-κB and Jun kinase pathways [166]. Secreted fizzled-related protein 1 (SFRP1), an inhibitor of Wnt signaling, has been identified as the target gene of EZH2 and is associated with the activation of RASFs.

### 5.2. Disordered DNA Methylation in MMP Genes in RA

Low levels of global DNA methylation have been demonstrated in both RA synovial tissues and RASFs [167]. Proliferating RASFs have been shown to be deficient in DNMT1 expression. The 5-azacytidine (5-azaC), an inhibitor of DNMTs, provides normal SFs with the activated phenotype of RASFs. The resulting DNA hypomethylation upregulates the expression of 186 genes, including MMP-1 and MMP-14. Although both DNMTs and TET enzymes might disrupt DNA methylation in RA, it remains unknown whether TET enzymes are involved in the pathogenesis of RA. Disordered DNA methylation-catalyzing enzymes may affect the expression of various genes, including MMP genes.

### 5.3. Disordered miRNA Expression in MMP Genes in RA

Microarray analysis has shown that both miR-155 and miR-146a are highly expressed in RASFs [168]. The overexpression of miR-155 represses MMP-3 expression and reduces the induction of MMP-1 and MMP-3 by Toll-like receptor (TLR) ligands and cytokines in RASFs. miR-155 possibly has a counter-regulatory role in the process of joint destruction in RA. miR-155 might function as a protective miRNA that locally suppresses MMP expression and thereby blocks excessive tissue damage by inflammation. The same group examined differentially expressed miRNAs in RASFs by screening 260 miRNAs [169]. As a result, miR-203 was found to be highly expressed in RASFs. As DNA demethylation with 5-azaC has been shown to upregulate miR-203 expression, this miRNA is regulated by DNA methylation. The overexpression of miR-203 significantly enhances MMP-1 production. Another microarray analysis demonstrated that miR-155 expression is increased and can be induced by TNF-α in RASFs [170]. The enforced expression of miR-155 decreases MMP-3 expression in RASFs. Inhibitor of κ light polypeptide gene enhancer in B-cells, kinase ε (IKBKE) increases the production of MMP-3 and MMP-13 by TNF-α, IL-1β and lipopolysaccharide (LPS) through c-Jun phosphorylation in RASFs [171]. IKBKE has been shown to be a target of miR-155. It has been suggested that miR-155 may be a protective factor against inflammation by attenuating the expression of IKBKE in RASFs. Another microarray analysis revealed that miR-19b is downregulated in RASFs [172]. The overexpression of miR-19b decreases the expression of MMP-3, and has been suggested to function as a negative regulator of inflammation in RA.

## 6. Conclusions

MMPs are produced by RASFs and play a crucial role in cartilage destruction in the inflamed joints in RA. Therefore, understanding the mechanisms regulating MMP expression is critical for developing RA therapeutics. Recently, substantial and accumulating data on epigenetic dysregulation has emerged in RA. Disorders of epigenetic regulation, including histone modifications, DNA methylation and miRNA signaling, are involved in the upregulation of MMP expression in RASFs. Further study is needed to elucidate the crosstalk among these different epigenetic mechanisms. It is hoped that advanced knowledge of the epigenetic mechanisms that control MMP gene activation in RASFs will provide a better understanding of the pathogenesis of RA and help develop new therapeutic strategies for this pernicious disease.

## Figures and Tables

**Table 1:** The matrix metalloproteinase (MMP) family.

Enzyme	Pseudonyms	Collagen Substrates	Additional Substrates
MMP-1	Collagenase-1	I, II, III, VII, VIII, X	Aggrecan, Gelatin
MMP-2	Gelatinase A	I, II, III, IV, V, VII, X, XI	Aggrecan, Elastin, Fibronectin, Gelatin, Laminin
MMP-3	Stromelysin-1	II, III, IV, IX, X, XI	Aggrecan, Elastin, Fibronectin, Gelatin, Laminin
MMP-4	Identified as MMP-3	-	-
MMP-5	Identified as MMP-2	-	-
MMP-6	Identified as MMP-3	-	-
MMP-7	Matrilysin	IV, X	Aggrecan, Elastin, Fibronectin, Gelatin, Laminin
MMP-8	Collagenase-2	I, II, III, V, VII, VIII, X	Aggrecan, Elastin, Fibronectin, Gelatin, Laminin
MMP-9	Gelatinase B	IV, V, VII, X, XIV	Aggrecan, Elastin, Fibronectin, Gelatin
MMP-10	Stromelysin-2	III, IV, V	Aggrecan, Elastin, Fibronectin, Gelatin, Laminin
MMP-11	Stromelysin-3	Aggrecan, Fibronectin, Laminin	-
MMP-12	Macrophage	IV	Elastin, Fibronectin, Gelatin, Laminin
MMP-13	Collagenase-3	I, II, III, IV	Aggrecan, Gelatin
MMP-14	MT1-MMP	I, II, III	Aggrecan, Elastin, Fibronectin, Gelatin, Laminin
MMP-15	MT2-MMP	Fibronectin, Gelatin, Laminin	-
MMP-16	MT3-MMP	-	-
MMP-17	MT4-MMP	Fibrin, Gelatin	-
MMP-18	Identified as MMP-19	-	-
MMP-19	RASI-1	IV	Aggrecan, Fibronectin, Gelatin, Laminin, COMP
MMP-20	Enamelysin	Aggrecan, Amelogenin, COMP	-
MMP-21	X-MMP	-	-
MMP-22	C-MMP	-	-
MMP-23	Identified as MMP-23	-	-
MMP-24	MT5-MMP	-	-
MMP-25	MT6-MMP	IV	Fibronectin, Gelatin, Laminin
MMP-26	Matrilysin-2	IV	Fibronectin, Gelatin
MMP-27	-	-	-
MMP-28	Epilysin	-	-

MT: Membrane-type; COMP: Cartilage oligomeric protein.
